# From wheat bran to equine gut: the *in vitro* fermentation dynamics of aleurone

**DOI:** 10.3389/fphys.2025.1644738

**Published:** 2025-11-11

**Authors:** Berit Boshuizen, Maarten Willems, Lorie De Maré, Guilherme Hosotani, Jean Eduardo De Oliveira, Benjamin Horemans, Carmen Vidal Moreno De Vega, Elisabeth-Lidwien J. M. M. Verdegaal, Catherine Delesalle

**Affiliations:** 1 Department of Translational Physiology, Infectiology and Public Health, Research Group of Comparative Physiology, Faculty of Veterinary Medicine, Ghent University, Merelbeke, Belgium; 2 Equine Hospital Wolvega, Oldeholtpade, Netherlands; 3 Cargill Research and Development Centre Europe, Vilvoorde, Belgium; 4 Equine Health and Performance Centre, School of Animal and Veterinary Sciences, Roseworthy Campus, University of Adelaide, Adelaide, SA, Australia

**Keywords:** aleurone, equine gastrointestinal tract, metabolism, metabolomics, hindgut microbiota, functional feed, time-resolved metabolite profiling, artificial intelligence modeling

## Abstract

**Introduction:**

Aleurone, a bioactive wheat bran component, has been shown to modulate host metabolism and gut microbiota, but its effects across different compartments of the equine gastrointestinal (GI) tract remain unclear. In this study, we aimed to characterize aleurone-derived metabolite profiles using an *in vitro* fermentation model with digesta from three equine GI compartments (jejunum, cecum, and colon).

**Methods:**

Three substrates (control feed, aleurone-containing feed, and pure aleurone) were fermented over 72 h, and targeted metabolomics was performed on 38 metabolites.

**Results:**

Significant substrate- and compartment-dependent effects were found for 21 metabolites. Aleurone-containing feed increased asparagine and threonine levels while reducing lactic acid, particularly in the cecum. In contrast, control feed showed the highest overall metabolite abundance, suggesting greater microbial accessibility. Time-resolved analyses revealed dynamic production–utilization patterns; isoleucine, for example, displayed a distinct peak–decay pattern in the colon. Carnitine increased over time across compartments, showing local production, especially in the cecum. Artificial intelligence-based classification models achieved >90% accuracy in distinguishing substrate types and revealed ferulic acid and indole acetic acid as key differentiators.

**Discussion:**

The findings suggest that aleurone’s structural matrix may influence metabolite release and microbial access, highlighting its functional role in modulating fermentation and overall host metabolism. In this study, we demonstrate that aleurone alters microbial fermentation and metabolite output in a time- and compartment-specific manner. These insights enhance our understanding of aleurone as a functional feed component in horses and provide a foundation for future dietary strategies targeting metabolic and gut health.

## Introduction

There is increasing scientific evidence that aleurone, a component derived from wheat bran, acts as a prebiotic, providing a substrate for the microbial community both in humans and several different animal species. Aleurone is the outermost living cell layer(s) of a cereal grain’s endosperm. These cells are rich in proteins and lipids, and during germination, they respond to gibberellins from the embryo by synthesizing and secreting hydrolytic enzymes (e.g., α-amylase and proteases) that mobilize the starch and proteins in the starchy endosperm. In milling, aleurone usually remains with the bran fraction. The aleurone layer can be found in the following grain types: wheat, rye, barley, oats, rice, maize (corn), sorghum, millets, and triticale (Bohm and Buri, 2004; Brouns et al., 2010; 2012; Fardet, 2010; Pekkinen et al., 2014; Quemeneur et al., 2020; Deng et al., 2021; Boshuizen et al., 2021; Boshuizen et al., 2025). Aleurone is considered a key contributor to the health benefits associated with whole grain consumption in humans. However, many consumers are discouraged by the coarse texture of whole grain products. To address this, the food industry is increasingly recognizing aleurone’s potential to enhance the fiber and nutrient content of foods without significantly altering their texture, and the milling industry has developed advanced separation techniques to isolate the aleurone layer from the bran fraction, yielding concentrates of differing purity—such as ASP1 and purer ASP2 (Roye et al., 2020; Lebert et al., 2022).

Moreover, the feed industry shows increasing interest in aleurone. It has been demonstrated that aleurone triggers multiple physiological effects across animal species, including, most recently, in pigs and horses (Quemeneur et al., 2020; Deng et al., 2021; Boshuizen et al., 2021; 2025). In pigs, aleurone supplementation has been shown to positively influence the feeding behavior, nitrogen metabolism, and energy partitioning. In particular, aleurone reduces the number of meals per day and decreases nitrogen retention, suggesting altered protein metabolism and nutrient utilization strategies (Le Gall et al., 2009; Quemeneur et al., 2020). Furthermore, in gestating sows, dietary inclusion of wheat aleurone improved postprandial satiety (via increased peptide YY and glucagon like peptide-1) and stress parameters (reduced salivary cortisol), and significantly lowered the stillbirth rate, indicating both metabolic and reproductive benefits (Quemeneur et al., 2020; Deng et al., 2021).

Both untrained and trained horses have been studied. In untrained horses, it was shown that daily feeding ≥200 g of aleurone (ASP1) significantly changed the glucose and insulin responses to a standardized meal; for example, the time to peak for both blood glucose and insulin increased, whereas the area under the curve (AUC) and maximum levels of glucose remained the same, and AUC_insulin_ and maximum insulin levels decreased. These study results suggest an increase in insulin sensitivity as a result of aleurone supplementation in untrained horses. In addition, aleurone-supplemented horses showed significant changes in the relative abundance in higher taxonomic levels of their fecal microbiome and decreased relative abundance of the following genera: *Roseburia*, *Shuttleworthia*, *Anaerostipes*, *Faecalibacter*, and *Succinovibrionaceae*. The most pronounced changes in relative abundance at the phylum level were observed in *Firmicutes* and *Verrucomicrobia* (downregulation), as well as in *Bacteroidetes* and *Spirochaetes* (upregulation) (Boshuizen et al., 2021; Boshuizen et al., 2025). This shift is often interpreted as beneficial, especially in the context of metabolic health, insulin sensitivity, or reduction in obesity-associated profiles. A high *Firmicutes*/*Bacteroidetes* ratio is often associated with metabolic dysregulation, so a shift toward more *Bacteroidetes* may imply improved gut efficiency or anti-inflammatory potential (Fitzgerald et al., 2020; Górniak et al., 2021).

Shifts in the composition of the gut microbiome can lead to changes in the metabolic fingerprint of the microbiome (Holmes et al., 2012; Proudman et al., 2015; Lloyd-Price et al., 2019; Zimmermann et al., 2019; Wilmanski et al., 2021; Boshuizen et al., 2021; 2025). Some of these metabolites have already been identified as having beneficial health effects. For example, it has been established that as a part of the dietary fiber fraction, aleurone is partly digested by the gut microbiota, which leads to the production of short-chain fatty acids (SCFAs) (i.e., acetate, butyrate, and propionate) (Glinsky et al., 1976; Cheng et al., 1987; Hughes et al., 2007). These SCFAs could modulate health benefits both locally at the level of the gastrointestinal (GI) tract (Bedford and Gong, 2018) and systemically, for example, through the amelioration of insulin sensitivity (Gao et al., 2009; Al-Lahham et al., 2010; Freeland et al., 2010). Other by-products in the fermentation of fibers using the gut microbiota include lactate, α-oxovalerate, aromatic amino acids (AAAs), and branched-chain amino acids (BCAAs). In conclusion, the results of the equine study supplementing aleurone to non-trained horses showed that aleurone blunts the postprandial glucose and insulin response and induces significant shifts in the fecal microbiome (Boshuizen et al., 2021; Boshuizen et al., 2025).

In trained Standardbred mares, aleurone further enhanced the insulin-sensitizing effects of exercise, as demonstrated by improved glucose effectiveness and lowered acute insulin response to glucose (AIRg) during intravenous glucose tolerance tests. Microbiome analysis revealed that training alone decreased *Pseudomonas*, a genus associated with dysbiosis, whereas aleurone supplementation reduced the relative abundance of inflammation-associated Desulfovibrio (Boshuizen et al., 2025). These findings further support a role for aleurone in modulating the metabolic–microbiome axis, referring to the dynamic and bidirectional relationship between the host’s metabolism and the composition and activity of its intestinal microbiota.

It is to be expected that aleurone’s prebiotic and metabolic regulatory roles are governed by species-specific digestive physiology. In particular, the extensive hindgut fermentation capacity found in horses may influence aleurone’s site of degradation/fermentation and absorption. Both *in vivo* and *in vitro* studies across different animal species have demonstrated that the bioavailability of the key components of the aleurone fraction, among which are ferulic acid and betaine, depends on how efficiently the organism releases them from the arabinoxylan-containing fiber matrix (Andreasen et al., 2001; Amrein et al., 2003; Zhao et al., 2003; Hughes et al., 2007; Neyrinck et al., 2008; Anson et al., 2009; Rosa et al., 2014; Keaveney et al., 2015; Bresciani et al., 2016; Boshuizen et al., 2021; Boshuizen et al., 2025). According to previous *in vitro* studies, there is evidence that bioaccessibility of the different aleurone components can be enhanced by the application of ultrafine grinding techniques and by the solubilization of cell-wall polysaccharides via enzymatic processing (Anson et al., 2009; Hemery et al., 2010; Calani et al., 2014; Pekkinen et al., 2014; Rosa et al., 2014; Aslam et al., 2018; Lebert et al., 2022). *In vivo*, it is expected that fermentation by gut microbiota during the gastrointestinal passage has an important impact on bioavailability of aleurone components. For this reason, an equine animal model was chosen for performing both *in vivo* and *in vitro* studies, with an extensive fermentation capacity in its hindgut (Mackie and Wilkins, 1988; Schoster et al., 2013). At this point, only a few *in vivo* studies have been performed, either in mice or in pigs (Neyrinck et al., 2008; Le Gall et al., 2009; Theil et al., 2011; Nørskov et al., 2013; Calani et al., 2014; Pekkinen et al., 2014; Rosa et al., 2014; Ingerslev et al., 2017; Quemeneur et al., 2019; Quemeneur et al., 2020; Deng et al., 2021; Sun et al., 2023), and, more recently, in horses (Boshuizen et al., 2021; Boshuizen et al., 2025).

It has been suggested that aleurone mediates its effects through a combined effect of factors, such as 1) the effect of aleurone and its components on feed texture and subsequent digestive processing, 2) the effect of aleurone and its components on metabolism, and 3) microbiome composition and metabolic output (Boshuizen et al., 2021).

Region-specific *in vitro* fermentation assays using inocula from different parts of the equine GI tract can pinpoint where aleurone is degraded and which metabolites are produced, thereby clarifying how it may affect host metabolism. These assays simulate intestinal conditions and quantify both the physical breakdown of the substrate and the chemical outputs. By comparing jejunal, cecal, and colonic inocula, they reveal when and where key nutrients are released and infer downstream cellular and systemic effects from the fermentative end-products. *In vitro* platforms also allow tight control of pH, temperature, redox state, and enzyme activity conditions difficult to standardize *in vivo* without invasive models, thereby providing mechanistic insight into aleurone–digestive interactions. As such, they serve as a complementary tool to generate hypotheses for subsequent validation in living animals (Mcclements et al., 2009; Bosch et al., 2017; Jeong et al., 2019; Leng et al., 2020).

We aimed to characterize and compare the *in vitro* fermentation/metabolic profiles of control feed, aleurone-containing feed, and purified aleurone (ASP1) using equine jejunum, cecum, and colon inocula in an *ex vivo* dynamic model. Our primary hypothesis (H1) was that aleurone-containing substrates yield distinct fermentation/metabolomic signatures relative to control feed, with effects most pronounced under hindgut (cecal/colonic) inocula compared with jejunal inoculum. In addition, the following secondary hypotheses were addressed in this study: (H2) the magnitude of aleurone effects follows purified aleurone ≥ aleurone feed > control; and (H3) a binary, supervised AI model can (i) classify aleurone vs. non-aleurone fermentation profiles above chance across compartments and (ii) identify candidate aleurone-specific biomarkers for future studies. For this purpose, a 3 × 3 factorial (substrate × compartment) *ex vivo* fermentation protocol was performed; metabolomic and physicochemical readouts enable direct substrate-by-compartment contrasts to test H1–H2. The same dataset feeds the AI classifier to test H3 and nominate features for follow-up work *in vivo* and in dietary applications.

## Materials and methods

*Experimental design:* the fermentation was evaluated over a period of 72 h, with a total of 27 fermentation bottles and one blank bottle. The experimental design included three substrates (control feed without aleurone, aleurone-containing feed, and pure aleurone) and three inocula (jejunal, cecal, and colonic digesta) collected from horses in the slaughterhouse, leading to a total of nine different treatment combinations, with three replicates each. Table 1 provides an overview of the study design. Supplementary Table S1 provides an overview of the macronutrient composition of both the control feed and aleurone-containing feed. Both diets were formulated to provide comparable energy and digestible protein levels (not identical; EWpa 0.701 vs. 0.708; DCP 85.6 vs. 83.4 g/kg), with the control diet slightly higher in starch and sugars (342 vs. 316 g/kg) (Supplementary Table S1). *Inoculum collection and transportation:* In brief, the insulated containers were pre-warmed by filling them with warm distilled water and remained closed until inoculum collection to maintain optimal temperature. During collection, efforts were made to minimize exposure of the intestinal content to temperature changes and oxygen. Immediately before sample collection, the containers were opened, and the warm water was discarded. Immediately after stunning and evisceration, horse digesta was collected from the end of the jejunum, cecum, and colon ascendens from each horse. Each container was filled with 500–1,000 mL of digesta (depending on the target compartment: jejunum versus, for example, cecum or colon), ensuring that it was filled to more than 90% of its volume to minimize oxygen exposure and pressure changes. “Stripping” of the intestinal segments was avoided. The inoculum was transported in a temperature-controlled transport incubator (37.5 °C) to the laboratory within 1 hour.

**TABLE 1:** Overview of the study design, detailing the specific combinations of substrates and GI compartments sampled for incubation.

Bottle	Substrate	Inoculum	Horse	Bottle	Substrate	Inoculum	Horse
1	Control feed	Jejunum	1	15	Aleurone-containing feed	Cecum	2
2	Control feed	Colon	1	16	Pure aleurone	Jejunum	2
3	Control feed	Cecum	1	17	Pure aleurone	Colon	2
4	Aleurone-containing feed	Jejunum	1	18	Pure aleurone	Cecum	2
5	Aleurone-containing feed	Colon	1	19	Control feed	Jejunum	3
6	Aleurone-containing feed	Cecum	1	20	Control feed	Colon	3
7	Pure aleurone	Jejunum	1	21	Control feed	Cecum	3
8	Pure aleurone	Colon	1	22	Aleurone-containing feed	Jejunum	3
9	Pure aleurone	Cecum	1	23	Aleurone-containing feed	Colon	3
10	Control feed	Jejunum	2	24	Aleurone-containing feed	Cecum	3
11	Control feed	Colon	2	25	Pure aleurone	Jejunum	3
12	Control feed	Cecum	2	26	Pure aleurone	Colon	3
13	Aleurone-containing feed	Jejunum	2	27	Pure aleurone	Cecum	3
14	Aleurone-containing feed	Colon	2				

*Fermentation setup:* at the laboratory, the collected inocula were promptly utilized for the *in vitro* fermentation experiments. Each treatment was incubated under anaerobic conditions at 37 °C, with samples collected from the fermentation setup at the initiation of the experiment (T_0_) and at subsequent time points (T_3_, T_6_, T_24_, T_48_, and T_72_). Additionally, a separate bottle, containing neither substrate, was included as a blank control.

The applied *in vitro* fermentation protocol was adapted from the study by Goering and Van Soest (1970). Throughout the procedure, all equipment and consumables involved in the handling or storage of samples were pre-warmed and maintained at 38 °C to preserve physiological conditions, with the exception of nitrogen (N_2_) used to flush the bottles to maintain anaerobic conditions. Substrates (control feed, aleurone-containing feed, and pure aleurone) were weighed, and 500 mg of each sample was added into 50 mL Ankom bottles. Subsequently, 35 mL of *in vitro* buffer solution (2 g NH_4_HCO_3_, 17.5 g NaHCO_3_, 2.86 g Na_2_HPO_4_, 3.1 g KH_2_PO_4_, 0.3 g MgSO_4_, and 0.25 mL 0.1% (w/v) resazurin brought to a final volume of 2 L with water) was added to each bottle. The ankom bottles were placed in an incubator shaker (Infors HT, model Ecotron, Bottmingen, Switzerland) and kept sealed with bottle caps.

The freshly collected inocula from the three GI sections (jejunum, cecum, and colon) were prepared by filtration through a single layer of cheesecloth and subsequently mixed with an *in vitro* buffer solution at a 1:2.5 ratio (inoculum:buffer). A volume of 15 mL of the prepared inoculum was precisely added to each designated bottle using a pipette.

Fermentation of the substrates was assessed at the initiation of the experiment (T_0_) and at subsequent intervals (T_3_, T_6_, T_24_, T_48_, and T_72_). At each time point, duplicate 1 mL samples were collected in Eppendorf tubes and stored at −80 °C until further analysis.

*Targeted metabolomics*: after collection, samples were centrifuged at 10,000 rpm at room temperature for 5 min. Then, 100 µL of each sample was diluted in 900 µL of 50:50 MiliQ-H_2_O/acetonitrile and again centrifuged at 10,000 rpm at room temperature for 5 min. After that, 700 µL of this solution was transferred to a high-performance liquid chromatography (HPLC) vial for analysis. Ultra-high-performance liquid chromatography (UHPLC, Thermo UltiMate 3,000, Thermo Fisher™ Scientific, Belgium) and quadrupole-orbitrap mass spectrometry (MS) (Thermo Q-Exactive Focus, Thermo Fisher Scientific™, Belgium) were performed using a 1.7 µm, 100 Å, 100 × 2.1 mm column (Kinetex PFP, Phenomenex, Belgium), MiliQ water, and 0.1% formic acid in channel A and acetonitrile and 0.1% formic acid in channel B. UHPLC-MS analysis and quantification were performed in positive and negative ion modes. Compound Discoverer (Thermo Fisher™ Scientific, Belgium) and Chromeleon (Thermo Fisher™ Scientific, Belgium) software were used to assess the metabolites in each sample. Each sample was analyzed in triplicate for the presence of a series of 38 metabolites, which were preselected based on the studies performed by Pekkinen et al. (2014), Rosa et al. (2014), and Bresciani et al. (2016). The selected metabolites were as follows: hippuric acid, methyl hippuric acid, leucine, isoleucine, valine, phenylalanine, phenyl lactate, tryptophan, tyrosine, histidine, alanine, glutamate, glutamine, taurine, asparagine, threonine, lysine, aspartate, citrate, malate, succinate, fumarate, betaine, NN-dimethylglycine, carnitine, butyrate, propionate, 3-hydroxy isobutyrate, α-oxovalerate, β-hydroxybutyrate, pyridoxine, urate, 5-hydroxyindoleacetate, N-methyldiphenylamine, trans-ferulic acid, indole acetate, lactate, and choline.

*Statistical analysis:* statistical analyses were performed using R software (version 4.0.2 (Team, 2020)). The linear mixed model was used to analyze the data using the lmer function from the “lme4” package (Bates et al., 2015). The model was fitted with restricted maximum likelihood (REML). Denominator degrees of freedom were computed using the Satterthwaite method. The model was specified as follows:

Yij = β0 + β1substrate + β2inoculum + β3time + β4substrate x inoculum + β5substrate x time + β6inoculum x time + bi + εij,

where Yij is the AUC for each metabolite, β0 is the overall mean, β1 is the fixed effect of the substrate, β2 is the fixed effect of inoculum (GI section), β3 is the fixed effect of time (incubation timepoint), β4 is the interaction between the substrate and inoculum, β5 is the interaction between the substrate and time, β6 is the interaction between the inoculum and time, bi is the random effect of individual horse, and εij is the residual error.

Tukey’s *post hoc* tests were performed to test for pairwise differences. All statistical tests were conducted at a significant level of α ≤ 0.05. The results were reported as mean estimates with their associated standard errors and p-values.

The following analyses were performed: time-independent substrate effects, time-independent compartment effects, time-dependent substrate effects per compartment, identification of time-dependent and time-independent substrate-specific metabolic profiles, time-dependent patterns in total significant metabolite abundance under different feed conditions across all three compartments taken together, and time-independent patterns in significant metabolite abundance under different feed conditions across all three compartments taken together.

*Application of a binary AI model*: in a second step, an AI model was applied to address the following questions: 1. Can an AI model be trained to classify horse samples accurately and assign them to the correct class using metabolomic data? 2. What are the most important metabolites for correctly assigning samples to their respective groups, by feeding following parameters to the AI model: time points: short (T_0_ to T_6_) and long time interval (T_24_ to T_72_); substrates: control feed, aleurone-containing feed, and pure aleurone; intestinal inoculum: jejunum, cecum, and colon.

The AI-based model hypothesis was developed using HoRIZON software.

The study involved the following parameters: time points: short (T_0_, T_3_, and T_6_) and long interval (T_24_, T_48_, and T_72_); substrates: control feed, aleurone-containing feed, and pure aleurone; intestinal inoculum: jejunum, cecum, and colon.

Data processing and model training were performed as follows: Data binarization: datasets were binarized to facilitate model training and evaluation. Classification of models: various classification models were tested to identify nonlinear relationships between metabolites and sample classification. Model evaluation: models were considered valuable if the P-value of the permutation score was ≤0.05 (indicating significance superior to random) and the AUC was at least 85% (indicating high model accuracy).

### Models tested

A total of 39 models were developed to compare different treatments. Table 2 provides an overview of the different AI models.

**TABLE 2:** Overview of the different AI models, detailing the specific combinations of substrates, gastrointestinal compartments, and time interval.

Model	Substrate	Compartment	Time interval	Model	Substrate	Compartment	Time interval
1	Control feed vs. aleurone-containing feed	All	Short	21	Aleurone-containing feed vs. pure aleurone	Colon	Short
2	Control feed vs. pure aleurone	All	Short	22	Control feed vs. aleurone-containing feed	Colon	Long
3	Aleurone-containing feed vs. pure aleurone	All	Short	23	Control feed vs. pure aleurone	Colon	Long
4	Control feed vs. aleurone-containing feed	All	Long	24	Aleurone-containing feed vs. pure aleurone	Colon	Long
5	Control feed vs. pure aleurone	All	Long	25	Control feed	All	Short vs. long
6	Aleurone-containing feed vs. pure aleurone	All	Long	26	Aleurone-containing feed	All	Short vs. long
7	Control feed vs. aleurone-containing feed	Jejunum	Short	27	Pure aleurone	All	Short vs. long
8	Control feed vs. pure aleurone	Jejunum	Short	28	Control feed vs. aleurone-containing feed	All	All
9	Aleurone-containing feed vs. pure aleurone	Jejunum	Short	29	Control feed vs. pure aleurone	All	All
10	Control feed vs. aleurone-containing feed	Jejunum	Long	30	Aleurone-containing feed vs. pure aleurone	All	All
11	Control feed vs. pure aleurone	Jejunum	Long	31	Aleurone-containing feed	Jejunum vs. cecum	Short
12	Aleurone-containing feed vs. pure aleurone	Jejunum	Long	32	Aleurone-containing feed	Jejunum vs. colon	Short
13	Control feed vs. aleurone-containing feed	Cecum	Short	33	Aleurone-containing feed	Cecum vs. colon	Short
14	Control feed vs. pure aleurone	Cecum	Short	34	Pure aleurone	Jejunum vs. cecum	Short
15	Aleurone-containing feed vs. pure aleurone	Cecum	Short	35	Pure aleurone	Jejunum vs. colon	Short
16	Control feed vs. aleurone-containing feed	Cecum	Long	36	Pure aleurone	Cecum vs. colon	Short
17	Control feed vs. pure aleurone	Cecum	Long	37	Control feed	Jejunum vs. cecum	Short
18	Aleurone-containing feed vs. pure aleurone	Cecum	Long	38	Control feed	Jejunum vs. colon	Short
19	Control feed vs. aleurone-containing feed	Colon	Short	39	Control feed	Cecum vs. colon	Short
20	Control feed vs. pure aleurone	Colon	Short				

Assessment of feature importance and metabolite selection was performed as follows:

Feature importance: the importance of each metabolite was assessed based on the loss of model quality when the metabolite was removed from the dataset. The higher the loss of quality, the higher the importance of the metabolite. Random variable: in all models, a random made-up variable (Random_variable) was included as a cutoff. Metabolites ranked below this variable were not considered significant. Top important metabolites: the most important metabolites were identified on each occasion and further analyzed for mathematical correlation. The study of correlations and network analysis was performed as follows: mathematical correlation: correlations between important metabolites were analyzed to determine whether they changed in the same or opposite directions. Network plots: network plots were created to visualize the relationships between metabolites. The size of each circle indicates the importance of the metabolite, whereas the lines represent the strength (line thickness) and direction of the correlation (blue for direct and red for inverse). A correlation threshold of at least |0.3| was set. Unconnected metabolites that did not meet this threshold are depicted in different colors.

## Results

### Time-independent substrate effect

The analysis of metabolites during the fermentation process revealed significant differences in 21 of the 38 pre-defined metabolites: alanine, asparagine, betaine, carnitine, choline chloride, ferulic acid, glutamic acid, glutamine, histidine, isoleucine, indoleic acid, lactic acid, leucine, lysine, malic acid, NN-dimethylglycine, phenylalanine, threonine, tryptophan, tyrosine, and valine based on the substrate and the gut compartment (i.e., jejunum, cecum, and colon).

**FIGURE 1:**
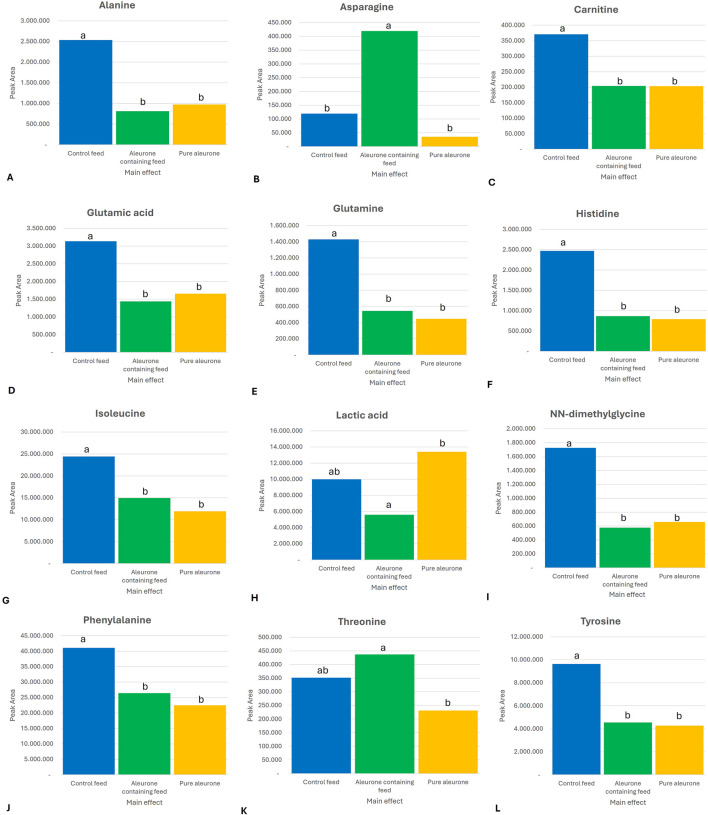
Bar graphs representing the significant substrate in the peak area for **(A)** alanine, **(B)** asparagine, **(C)** carnitine, **(D)** glutamic acid, **(E)** glutamine, **(F)** histidine, **(G)** isoleucine, **(H)** lactic acid, **(I)** NN-dimethylglycine, **(J)** phenylalanine, **(K)** threonine, and **(L)** tyrosine. The letters (a, ab, and b) above each bar represent the significance between the substrates. If two substrates have the same letter above, it means there is no significant difference between these two substrates for that specific metabolite. For example, for alanine, the control feed is significantly different from that of the aleurone-containing feed and the pure aleurone. However, there is no significant difference between the aleurone-containing feed and pure aleurone.

The most important results for time-independent substrate effect across all GI compartments for aleurone-containing feed compared to control feed were a significant increase in the levels of asparagine (P < 0.0001) and threonine (P = 0.000). Furthermore, there was a reduction in lactic acid (P = 0.002). In the group of pure aleurone, the overall metabolite abundance was lower than that of both the aleurone-containing feed and the control feed groups. The control feed group showed the highest abundance of alanine (P < 0.0001), carnitine (P < 0.0001), glutamic acid (P < 0.0001), glutamine (P < 0.0001), histidine (P < 0.0001), isoleucine (P < 0.0001), NN-dimethylglycine (P < 0.0001), phenylalanine (P < 0.0001), and tyrosine (P = 0.0001). Figure 1 shows the metabolites with different levels depending on the substrate type (control feed, aleurone-containing feed, and pure aleurone).

### Time-independent compartment effect

**FIGURE 2:**
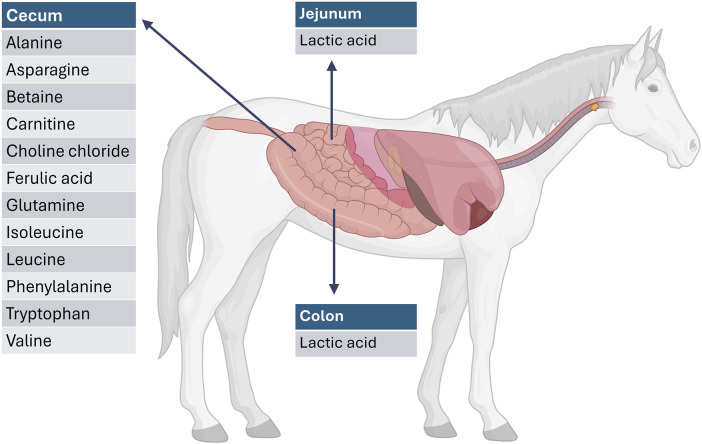
Representation of significantly elevated metabolites per GI compartment compared with the other GI compartments. For example, the metabolites listed at the level of the cecum are significantly increased when compared to the jejunum and the colon. Created in BioRender. Willems, M. (2025) https://BioRender.com/dzzz9ae.

**FIGURE 3:**
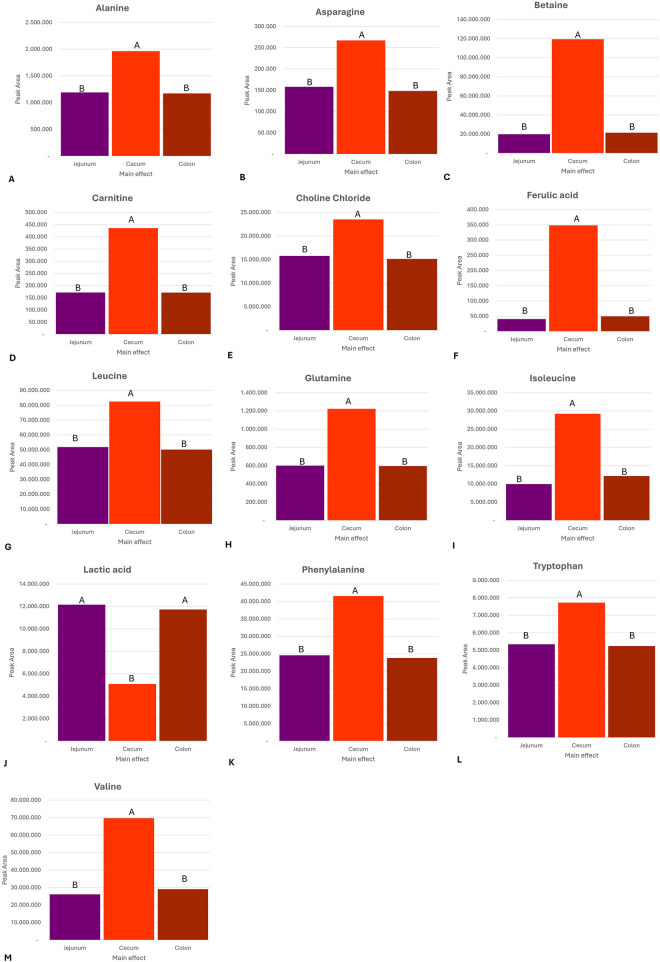
Bar graphs representing the compartment effect, across the three substrates (control feed, aleurone-containing feed, and pure aleurone), on the abundance in the peak area of **(A)** alanine, **(B)** asparagine, **(C)** betaine, **(D)** carnitine, **(E)** choline chloride, **(F)** ferulic acid, **(G)** leucine, **(H)** glutamine, **(I)** isoleucine, **(J)** lactic acid, **(K)** phenylalanine, **(L)** tryptophan, and **(M)** valine. The letters (A and B) above each bar represent the significance between the compartments (e.i. jejunum, cecum, and colon); the same letter means there is no significant difference between each other. If two substrates have the same letter above, it means there is no significant difference between these two GI compartment for that specific metabolite. For example, for alanine, the cecum is significantly different from that of the jejunum and the colon. However, there is no significant difference between the jejunum and the colon.

The metabolite profiles varied significantly across the different GI compartments. Figure 2 provides a graphical overview of the significantly elevated metabolites for each GI compartment compared to the other GI compartments. This is to be expected as the different GI compartments have different microbiome compositions. In both the jejunum and colon, lactic acid (P = 0.002) was significantly higher than that in the cecum. This is consistent with anaerobic activity in these compartments. In the cecum, there were elevated levels of alanine (P < 0.0001), betaine (P < 0.0001), asparagine (P = 0.019), carnitine (P < 0.0001), choline chloride (P = 0.000), ferulic acid (P = 0.000), glutamine (P = 0.004), isoleucine (P < 0.0001), leucine (P = 0.0001), phenylalanine (P < 0.0001), tryptophan (P < 0.0001), and valine (P < 0.0001). These are indicative of an anaerobic fermentation process. Figure 3 provides an overview of the compartment effect, across the three substrates (control feed, aleurone feed, and pure aleurone).

### Substrate × compartment interactions

**FIGURE 4:**
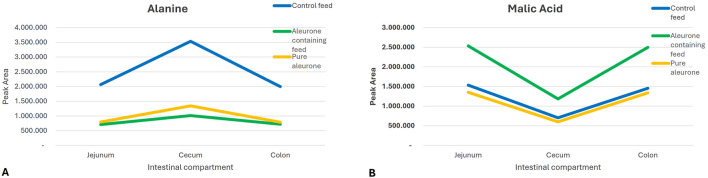
Graphs representing the differences in the peak area of **(A)** alanine and **(B)** malic acid for each substrate (control feed, aleurone-containing feed, and pure aleurone) used in each GI compartment.

The interaction between the substrate and the GI compartment indicated that the effect of the feed type on the metabolite production varied across the different sections of the gut. It suggests a compartment-specific utilization or production pattern of metabolites. The metabolites alanine (P = 0.038) and malic acid (P = 0.013) showed a significant interaction of production and utilization in different GI compartments. Alanine was more abundant in the cecum than in the other two compartments under control feed. In contrast to this, malic acid showed an opposite pattern. It was more abundant in the jejunum and colon than in the cecum under aleurone-containing feed and pure aleurone conditions (Figure 4).

### Time-dependent substrate effect by the compartment

**FIGURE 5:**
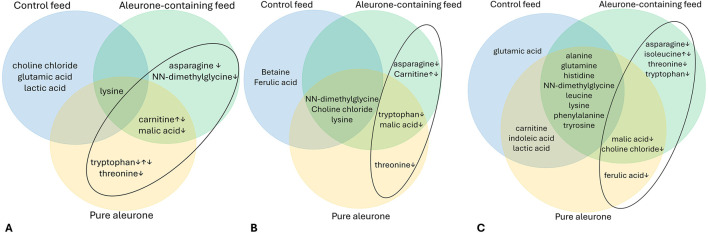
Venn diagram for the jejunum **(A)**, the cecum **(B)**, and the colon **(C)**, depicting the important time-dependent metabolites for each substrate (control feed, aleurone-containing feed, and pure aleurone). The ↓ and ↑ show the concentration trend over time (T_0_–T_72_). In the ellipse, the metabolites for aleurone-containing feed and pure aleurone are highlighted.

Figure 5 visually represents the relationships between the significant metabolite changes and the substrate for the jejunum (5A), cecum (5B), and colon (5C).

In the jejunum (Figure 5A), with aleurone-containing feed, there was a significant change over the time period T_0_–T_72_ for five metabolites: asparagine (P < 0.001), carnitine (P < 0.001), lysine (P < 0.001), malic acid (P < 0.001), and NN-dimethylglycine (P < 0.001). For the pure aleurone substrate, there was a significant change in five metabolites: carnitine (P = 0.038), lysine (P < 0.001), malic acid (P < 0.001), threonine (P = 0.006), and tryptophan (P = 0.032). In the control feed group, there was a significant change in four metabolites: choline chloride (P = 0.004), glutamic acid (P = 0.002), lactic acid (P = 0.001), and lysine (P = 0.019). The time lapses of up- or downregulation of these important metabolites for both aleurone-containing feed and pure aleurone is further discussed below (Figure 5C).

In the cecum (Figure 5B), with aleurone-containing feed, there were significant changes in seven metabolites along the time period T_0_–T_72_: asparagine (P < 0.001), carnitine (P < 0.001), choline chloride (P = 0.016), lysine (P < 0.001), NN-dimethylglycine (P < 0.001), tryptophan (P = 0.010), and malic acid (P = 0.005). With pure aleurone, this resulted in a significant change in six metabolites: choline chloride (P = 0.021), lysine (P < 0.001), malic acid (P < 0.001), NN-dimethylglycine (P = 0.010), threonine (P = 0.037), and tryptophan (P = 0.002). For the control feed, significant time changes were observed in five metabolites: betaine (P = 0.002), choline chloride (P = 0.004), ferulic acid (P = 0.037), lysine (P = 0.030), and NN-dimethylglycine (P = 0.017).

Finally, the most different metabolite changes over the time period T_0_–T_72_ were observed in the colon (Figure 5C). With aleurone-containing feed, a significant change was observed in 14 different metabolites: alanine (P < 0.001), asparagine (P = 0.022), choline chloride (P < 0.001), glutamine (P = 0.011), histidine (P < 0.001), isoleucine (P = 0.002), leucine (P < 0.001), lysine (P < 0.001), malic acid (P < 0.001), NN-dimethylglycine (P < 0.001), phenylalanine (P = 0.001), threonine (P < 0.001), tryptophan (P = 0.005), and tyrosine (P < 0.001). Pure aleurone resulted in a significant change in 14 metabolites: alanine (P < 0.001), carnitine (P = 0.017), choline chloride (P < 0.001), ferulic acid (P = 0.049), glutamine (P = 0.001), histidine (P < 0.001), indoleic acid (P = 0.027), lactic acid (P = 0.035), leucine (P = 0.005), lysine (P < 0.001), malic acid (P < 0.001), NN-dimethylglycine (P = 0.003), phenylalanine (P = 0.004), and tyrosine (P < 0.001). Finally, with control feed, there was a significant change in 12 metabolites: alanine (P < 0.001), carnitine (P < 0.001), glutamic acid (P < 0.001), glutamine (P = 0.006), histidine (P < 0.001), indoleic acid (P = 0.005), lactic acid (P = 0.013), leucine (P = 0.024), lysine (P = 0.002), NN-dimethylglycine (P = 0.004), phenylalanine (P = 0.002), and tyrosine (P < 0.001).

**FIGURE 6:**
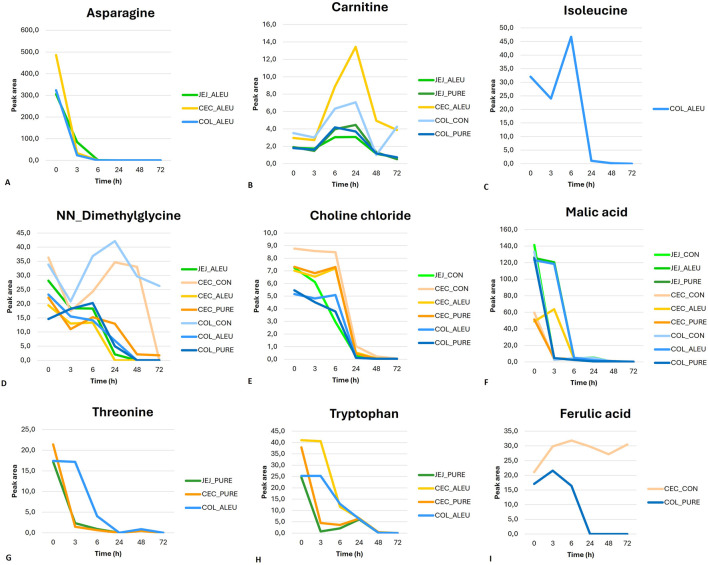
Time-course of metabolite abundancy for the different GI compartments is represented by different colors [(green for jejunum (JEJ), orange for cecum (CEC), and blue for colon (COL)], and substrates are represented by different color shades [light for control feed (CON), medium for aleurone-containing feed (ALEU), and dark for pure aleurone (PURE)].

The time trends of the main metabolites for aleurone-containing feed and pure aleurone revealed that for the aleurone feed, asparagine (Figure 6A) in all the GI compartments showed a sharp decrease until T_3_, followed by a less pronounced decrease until it disappeared at T_6_. Carnitine (Figure 6B) showed, at the level of the cecum with aleurone-containing feed and in the jejunum with both the aleurone-containing feed and pure aleurone, a peak at T_24_, a sharp decrease at T_48_, and a further gradual decrease thereafter. The time-dependent changes for isoleucine (Figure 6C) in the colon with aleurone-containing feed showed a decrease at T_3_, evolving into a peak at T_6_ before decreasing sharply at T_24_. In the jejunum with aleurone-containing feed, NN-dimethylglycine (Figure 6D) decreased until T_3_, remained stable until T_6_, and then decreased sharply until T_48_. In the colon, choline chloride (Figure 6E) with aleurone-containing feed showed a peak at T_6_ and subsequently decreased until T_24_. With pure aleurone, it began to decrease gradually, and at T_24_, the decrease became more pronounced. Malic acid’s (Figure 6F) abundance time profile is comparable for the jejunum and colon with aleurone-containing feed; it decreased gradually until T_3_ and then decreased sharply until T_6_. With pure aleurone, the jejunum and colon are also comparable; it decreased sharply until T_3_ and then decreased more gradually. In the cecum with aleurone-containing feed, it showed a peak at T_3_ and then decreased until T_6_; with pure aleurone, it decreased sharply until T_3_ and then more gradually thereafter. In the colon with aleurone-containing feed, threonine abundance (Figure 6G) decreased strongly from T_3_ to T_24_ and showed a small peak at T_48_. In the jejunum and cecum with pure aleurone, it decreased sharply until T_3_ and then more gradually until T_24_, followed by a small peak at T_48_. In the colon with aleurone-containing feed, tryptophan (Figure 6H) began decreasing at T_3_ to disappear at T_48_. At the level of the cecum with aleurone-containing feed, it started decreasing sharply at T_3_ and more gradually from T_6_ to T_48_. With pure aleurone, it decreased sharply to T_3_, gradually to T_6_, increased to a small peak at T_24_, and finally decreased until T_48_. In the jejunum with pure aleurone, it showed a strong decrease until T_3_ and started gradually to increase to reach a peak at T_24_. Finally, in the colon with pure aleurone, ferulic acid (Figure 6I) showed a peak at T_3_, with a decrease until T_24_.

### Long-term vs. short-term metabolite dynamics

The results of the effect of time between a short time interval (T_0_–T_6_) vs. a long time interval (T_24_–T_72_) on the metabolite changes over all the GI compartments showed a significant change in 15 metabolites: alanine (P < 0.001), asparagine (P < 0.001), betaine (P = 0.004), carnitine (P < 0.001), choline chloride (P = 0.008), ferulic acid (P < 0.001), glutamic acid (P < 0.001), glutamine (P = 0.002), histidine (P < 0.001), lysine (P = 0.006), malic acid (P < 0.001), NN-dimethylglycine (P < 0.001), threonine (P = 0.001), tryptophan (P < 0.001), and tyrosine (P < 0.001).

In general, these metabolites showed higher concentrations during the short time interval than during the long time interval, except for carnitine, which was present at a higher concentration during the long time interval.

### AI model insights

After running the AI model across all 39 structured models, the following models **(M)** with significant results of P ≤ 0.05 and with accuracy above 85% were obtained: (M1) control feed vs. aleurone-containing feed, short time (T_0_–T_6_), all compartments (accuracy = 99%; P = 0.0015); (M2) control feed vs. pure aleurone, short time, all compartments (accuracy = 98%; P = 0.0020); (M3) aleurone-containing feed vs. pure aleurone, short time, all compartments (accuracy = 96%; P = 0.0020); (M4) control feed vs. aleurone-containing feed, long time (T_24_–T_72_), all compartments (accuracy = 92%; P = 0.0075); (M5) control feed vs. pure aleurone, long time, all compartments (accuracy = 94%; P = 0.0065); (M28) control feed vs. aleurone-containing feed, complete time interval, all compartments (accuracy = 96%; P = 0.0005); and (M29) control feed vs. pure aleurone, complete time interval, all compartments (accuracy = 95%; P = 0.0005).

Each of these models provides a list of the most important metabolites, in a descending order of importance, and a network analysis of the correlation between the metabolites.

**FIGURE 7:**
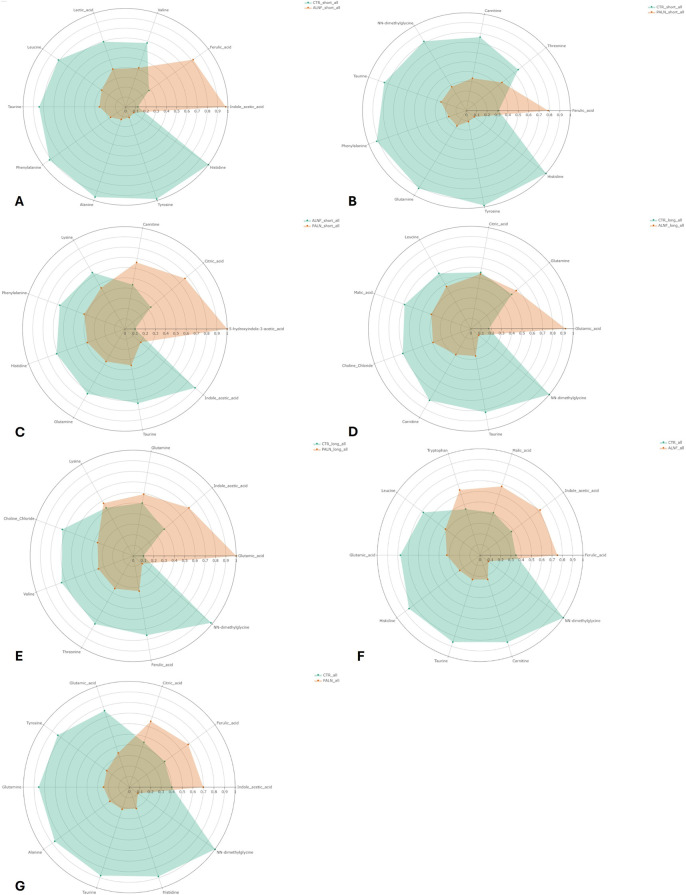
Radar diagrams of the metabolic profile of the AI models. Each colored area represents the relative concentration of the metabolite in the respective group. The distance from the center of the chart indicates the magnitude of the value; further out means higher concentration. Model 1 **(A)** green for control feed and red for aleurone-containing feed. This indicates a higher concentration of indole acetic acid and ferulic acid with aleurone-containing feed compared with the control feed. Model 2 **(B)** green for control feed and red for pure aleurone. This indicates a higher concentration of ferulic acid with pure aleurone compared with the control feed. Model 3 **(C)** green for aleurone-containing feed and red for pure aleurone. This indicates a higher concentration of 5-hydroxyindole-3-acetic, citric acid, and carnitine with pure aleurone compared with aleurone-containing feed. Model 4 **(D)** green for control feed and red for aleurone-containing feed. This indicates a higher concentration of glutamic acid and glutamine with aleurone-containing feed compared to the control feed. Model 5 **(E)** green for control feed and red for pure aleurone. This indicates a higher concentration of glutamic acid, indole acetic acid, glutamine, and lysine with pure aleurone compared with the control feed. Model 28 **(F)** green for control feed and red for aleurone-containing feed. This indicates a higher concentration of ferulic acid, indole acetic acid, malic acid, and tryptophan with aleurone-containing feed compared with the control feed. Model 29 **(G)** green for control feed and red for pure aleurone. This indicates a higher concentration of indole acetic acid, ferulic acid, and citric acid with pure aleurone compared with the control feed.

**FIGURE 8:**
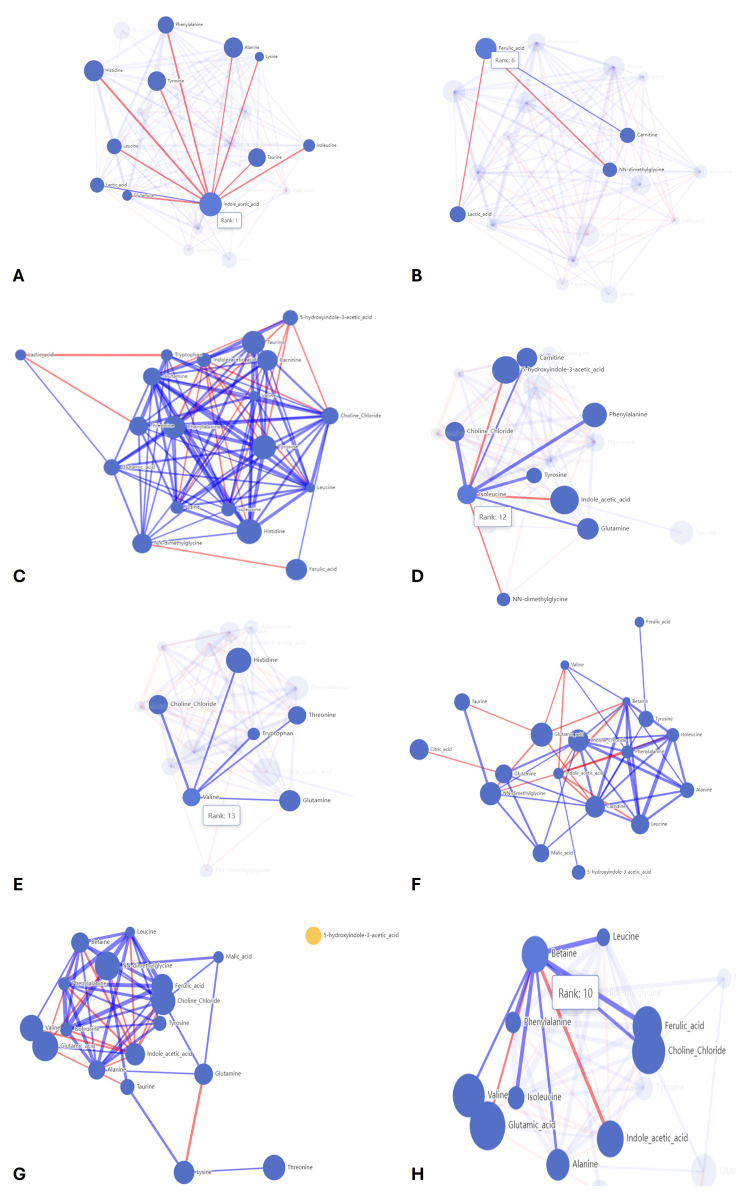
Correlation networks of the AI models. The size of the node represents the importance of the metabolite in the model. The lines represent positive (blue) and negative (red) correlations between metabolites. The width of the lines represents the strength of the correlation. **(A)** Correlation network for model 1, highlighting the correlations for indole acetic acid and **(B)** for ferulic acid. **(C)** Correlation network for model 2. **(D)** Correlation network for model 3, highlighting the correlations for isoleucine and **(E)** valine. **(F)** Correlation network for model 4. **(G)** Correlation network for model 5 highlighting the correlation for **(H)** betaine.

AI model 1 compared the control feed with aleurone-containing feed over a short time interval. This model resulted in 10 important metabolites: indole acetic acid, histidine, alanine, tyrosine, taurine, ferulic acid, phenylalanine, valine, leucine, and lactic acid. The aleurone-containing feed metabolic profile was different from that of the control, with a higher abundance of indole acetic acid and ferulic acid. An overview of the metabolic profile can be found in Figure 7A. The network analysis (Figures 8A,B) of this model showed a positive correlation between lactic acid and indole acetic acid but a negative correlation with most amino acids (phenylalanine, histidine, leucine, glutamine, lysine, alanine, tyrosine, isoleucine, and taurine). Furthermore, ferulic acid had a positive correlation with carnitine but a negative correlation with lactic acid and NN-dimethylglycine.

Model 2 compared control feed with pure aleurone for a short time interval, which resulted in eight important metabolites, namely, histidine, tyrosine, taurine, phenylalanine, ferulic acid, carnitine, NN-dimethylglycine, and threonine. Comparing the metabolic profiles (Figure 7B) of pure aleurone and the control, pure aleurone had a higher abundance of only ferulic acid. This was positively correlated to choline and negatively correlated to NN-dimethylglycine (Figure 8C).

Model 3 compared aleurone-containing feed with pure aleurone for a short time interval, which resulted in five important metabolites, namely, indole acetic acid, 5-hydroxyindole-3 acetic, lysine, histidine, and phenylalanine. The pure aleurone metabolic profile (Figure 7C) was associated with a higher abundance of 5-hydroxyindole-3- acetic than that of the aleurone-containing feed. The metabolic profile (Figure 7C) of the aleurone feed was associated with a higher abundance of histidine, phenylalanine, lysine, and indoleic acid. The network metabolite analysis (Figure 8D) showed a negative correlation between 5-hydroxyindole-3-acetic and isoleucine. Choline was positively correlated with both isoleucine and valine.

In model 4, the control feed was compared with the aleurone-containing feed for a long time interval. This resulted in five important metabolites: glutamic acid, NN-dimethylglycine, choline chloride, carnitine, and citric acid. The metabolic profile (Figure 7D) of aleurone-containing feed was associated with a higher abundance of glutamic acid and glutamine than that of the control group, with a higher abundance of NN-dimethylglycine, carnitine, choline chloride, and citric acid. Figure 8F provides the metabolic network for model 4.

Model 5 compared control feed with pure aleurone for a long time interval. This led to seven important metabolites: glutamic acid, NN-dimethylglycine, choline chloride, valine, threonine, ferulic acid, and lysine. The metabolic profile (Figure 7E) for pure aleurone resulted in higher abundance of glutamic acid, indole acetic acid, glutamine, and lysine, whereas that of control feed was associated with a higher abundance of NN-dimethylglycine, ferulic acid, threonine, valine, and choline chloride.

The metabolic network (Figures 8G,H) showed a positive correlation between glutamine and alanine, choline, and malic acid. However, it was negatively correlated to lysine. Betaine (Figure 8H) was negatively correlated to glutamic acid and indole acetic acid.

Model 28 compared control feed and aleurone-containing feed over all the GI compartments and the entire time interval. This resulted in 10 important metabolites: glutamic acid, indole acetic acid, NN-dimethylglycine, ferulic acid, carnitine, taurine, histidine, malic acid, leucine, and tryptophan. The metabolic profile (Figure 7F) of aleurone feed had a higher abundance of ferulic acid, indole acetic acid, malic acid, and tryptophan than that of the control feed, which had a higher abundance of NN-dimethylglycine, carnitine, taurine, histidine, glutamic acid, and leucine.

Model 29 compared control feed with pure aleurone over all the GI compartments and the entire time interval. This resulted in 10 important metabolites, namely, glutamic acid, taurine, histidine, ferulic acid, NN-dimethylglycine, tyrosine, glutamine, alanine, citric acid, and indole acetic acid. The metabolite profile (Figure 7G) of pure aleurone showed a higher abundance of indole acetic acid, ferulic acid, and citric acid. Compared to the metabolic profile of the control feed, higher abundances of NN-dimethylglycine, histidine, taurine, alanine, glutamine, tyrosine, and glutamic acid were observed.

## Summary

Across both time intervals together, significant changes were observed in the metabolites ferulic acid and indole acetic acid for both aleurone-containing feed and pure aleurone. Citric acid changed significantly only with the pure aleurone substrate.

Within the short time interval, a significant change occurred for metabolite ferulic acid in both aleurone-containing feed and pure aleurone. For indole acetic acid, this change occurred in aleurone-containing feed, whereas for citric acid, it occurred only with pure aleurone.

The long time interval showed, for both aleurone-containing feed and pure aleurone, a change in the metabolites (glutamine and glutamic acid). Indole acetic acid changed significantly only with pure aleurone.

## Discussion

In this study, we provide clear evidence that aleurone and aleurone-containing feed exert compartment- and time-dependent effects on microbial fermentation and metabolite production in the equine gastrointestinal tract. These findings are consistent with previous *in vivo* studies showing shifts in microbial composition and improved insulin sensitivity following aleurone supplementation in horses (Boshuizen et al., 2021; Boshuizen et al., 2025). The present data further specify that such effects are not uniform across the gut but are most pronounced in the cecum and colon, which are recognized as key fermentation sites (Dougal et al., 2013; Reed et al., 2021).

Within this context, several mechanistic explanations can be considered. The reduction in lactic acid under aleurone-containing feed, for example, may indicate enhanced lactate utilization by cecal microbes such as *Megasphaera elsdenii* or *Veillonella* spp., taxa known to maintain hindgut pH stability (Biddle et al., 2013; Louis et al., 2022). Similarly, the lower overall metabolite abundance observed with pure aleurone likely reflects its restricted nutrient profile, which limits broad microbial activity. At the same time, the structural matrix of aleurone may encapsulate fermentable substrates and modulate their release, delaying microbial access and contributing to the time-resolved patterns observed (Pekkinen et al., 2014; Lebert et al., 2022).

### Time-independent substrate effect

Aleurone-containing feed led to increased levels of asparagine and threonine and a reduction in lactic acid compared to control feed. The reduction in lactic acid may suggest improved fermentation efficiency or alternative microbial metabolic routes, which could be beneficial in maintaining hindgut pH stability (Biddle et al., 2013; Dicks et al., 2014). The lower overall metabolite abundance in the pure aleurone group likely results from its limited nutrient spectrum, which is insufficient to support broad microbial activity. Interestingly, the control feed led to the highest overall metabolite abundance. One explanation may lie in the physical structure of aleurone, which can encapsulate or slow the release of fermentable substrates, leading to a different fermentation output time profile when compared to aleurone-containing feed or control feed (Pekkinen et al., 2014; Lebert et al., 2022).

Table 3 provides an overview to explain why higher metabolic output values were observed for control feed when compared to the other two substrates (aleurone-containing feed or pure aleurone).

**TABLE 3:** An overview to explain why higher metabolic output values were observed for control feed when compared to the other two substrates (aleurone-containing feed or pure aleurone).

	Substrate for microbial growth	Source of metabolites	Scaffolding potential	Potential metabolite from microbial degradation
Control feed	+	+	+	+++
Aleurone-containing feed	++	++	++	++
Pure aleurone	+++	+++	+++	+

### Time-independent compartment effect

The cecum displayed significantly higher levels of various metabolites than the jejunum and colon, confirming its role as a major fermentation site in horses. This finding aligns with that of the previous literature, emphasizing the cecum’s anaerobic environment and rich microbial diversity. Lactic acid levels were higher in the jejunum and colon but low in the cecum, suggesting active lactate-utilizing microbes in the latter. Taxa such as *M. elsdenii* and *V.* spp., known for lactate consumption, may play a role. The metabolic output differences across compartments underline the importance of compartment-specific evaluation in equine digestive studies.

Although the colon, particularly the ventral and dorsal colon, contributes significantly to the fermentation process (Sneddon et al., 2006; Dougal et al., 2013; Reed et al., 2021), the cecum is often highlighted as the major site for microbial fermentation, with a significant role in the breakdown of dietary fibers and production of volatile fatty acids (Brandi and Furtado, 2009; Fujimori, 2021). The cecum is particularly important for hosting a variety of microorganisms that initiate the fermentation, whereas the colon continues this process, further breaking down the digesta (Dougal et al., 2013; Reed et al., 2021). The lower levels of lactic acid in the cecum suggest a possible metabolic pathway where lactic acid produced in the jejunum is utilized by cecal microbes for further fermentation, potentially contributing to the production of other beneficial metabolites such as SCFAs (Al Jassim et al., 2005; Mach et al., 2021; Louis et al., 2022). It has been described that taxa related to *M. elsdenii* and *Veillonella montpellierensis* are known to utilize lactate, reducing its concentration in the large intestine (Biddle et al., 2013). This aligns with the anaerobic conditions of the cecum, which favors the growth of microbes capable of metabolizing lactic acid. It is important to note that the control diet contained slightly more readily fermentable carbohydrate than the aleurone diet (starch + sugars ∼342 vs. 316 g/kg) (Supplementary Table S1), which could have induced a higher lactic acid production. However, when translating this to the *in vivo* study (Boshuizen et al., 2025), the magnitude is well below doses typically associated with pronounced hindgut lactate accumulation or large insulin excursions. Any effect of this small residual difference is likely minimal and does not change the interpretation of the lactate results. The observed differences are more readily explained by aleurone’s matrix properties and compartment-specific microbial responses.

### Time-dependent substrate effect by the compartment

Fermentation kinetics revealed time-specific dynamics in metabolite abundance. Several metabolites peaked early (T_3_ or T_6_) and declined, indicative of microbial utilization after initial substrate breakdown. These temporal profiles varied by GI compartment and substrate, highlighting localized microbial activity and metabolic specialization. The colon displayed the most dynamic fermentation profile, consistent with its diverse microbiota and extended digesta retention time.

Most metabolites significantly changed in the colon, followed by the cecum and jejunum. This is in agreement with previous studies suggesting that the concentration of volatile fatty acids increases significantly from the cecum to the colon (Miyaji et al., 2008) and the microbial population in the colon is distinct and more diverse than that of the cecum, contributing to a more extensive fermentation process (Dougal et al., 2013; Reed et al., 2021).

Isoleucine, an amino acid relevant for muscle metabolism in exercising horses, showed compartment-specific production, particularly in the colon under aleurone-containing feed, suggesting microbial biosynthesis (Zhuang et al., 2025).

The sustained presence or delayed peak of certain metabolites may be influenced by aleurone’s unique 3D structure. This scaffold-like matrix could physically trap nutrients and release them more slowly, affecting both substrate availability and microbial access. Similar mechanisms have been proposed in the field of bioengineered gut scaffolds and could explain some of the metabolic trends observed (Orlando et al., 2012; Lefebvre et al., 2015).

### Long-term vs. short-term metabolite dynamics

Most metabolites were more abundant in early time points (T_0_–T_6_), except carnitine, which increased over time and was particularly elevated in the cecum. This aligns with the known microbial synthesis of carnitine and highlights the cecum’s role in producing compounds involved in lipid metabolism and energy balance (Dambrova et al., 2022). Aleurone did not significantly alter carnitine levels, suggesting that its effects may be substrate- or compartment-specific.

### AI models

The application of AI modeling identified key metabolites that distinguish between substrates. Ferulic acid and indole acetic acid emerged as central differentiators for aleurone-containing feed and pure aleurone, respectively. Other metabolites such as glutamine, glutamic acid, and citric acid were also identified, reinforcing the capacity of aleurone to modulate amino acid and energy-related pathways. The high predictive accuracy of these models supports their use in future biomarker discovery and functional feed evaluation.

Static *in vitro* systems, although economical and high-throughput, lack the physiological dynamics of digestion such as peristalsis, enzyme gradients, and mucosal interactions. Future studies may benefit from coupling static fermentation with dynamic *in vitro* digestion models to improve physiological relevance (Sensoy, 2021). In contrast, dynamic *in vitro* models represent more accurately physiological conditions within the digestive system and can be either mono- or multi-compartmental (Guerra et al., 2012; Dupont et al., 2019; Liu et al., 2019; Sensoy, 2021). These models can simulate changes in pH, enzyme secretion, peristaltic forces, and microbial fermentation, proving a more comprehensive understanding of digestive processes (Liu et al., 2019; Sensoy, 2021).

### Linking metabolite profiles to microbial taxa across *in vitro* and *in vivo* aleurone studies

The *in vitro* fermentation trial identified several key metabolites, including ferulic acid, indole acetic acid, asparagine, threonine, and reduced lactate, which provide strong clues about the gut microbial taxa likely to be involved. Ferulic acid release is typically associated with members of the *Bacteroidetes* and *Firmicutes* (notably Lachnospiraceae and Ruminococcaceae), which harbor feruloyl esterases capable of liberating bound phenolics. Indole acetic acid derives from microbial tryptophan metabolism, often linked to *Clostridiales* and certain *Bacteroides* species. The observed reduction in lactate with aleurone-containing feed is consistent with enhanced utilization by lactate-consuming taxa such as *M. elsdenii* and *V.* spp., taxa known to stabilize hindgut pH. When aligned with the *in vivo* oral dosing trial (Boshuizen et al., 2021), where aleurone supplementation shifted the fecal microbiome toward higher *Bacteroidetes* and *Spirochaetes*, with a concomitant decrease in *Firmicutes* and *Verrucomicrobia*, these metabolite patterns suggest that aleurone promotes a microbial ecosystem enriched in fibrolytic- and phenolic-metabolizing phyla. The training study (Boshuizen et al., 2025) further supports this interpretation: aleurone supplementation reduced inflammation-associated *Desulfovibrio* and correlated positively with Ruminococcaceae and *Victivallis*, taxa linked to improved insulin sensitivity. Taken together, the concordance of metabolite profiles and microbiome shifts across *in vitro* and *in vivo* settings indicates that aleurone likely fosters a hindgut community where *Bacteroidetes*, Ruminococcaceae, and lactate-utilizing taxa are promoted, underpinning the observed improvements in metabolic flexibility and insulin sensitivity.

## Conclusion

In this study, we confirm that aleurone alters fermentation patterns and metabolite production in a compartment-specific and time-dependent manner. The metabolic effects were more pronounced with aleurone-containing feed than with pure aleurone, underscoring the importance of matrix interactions and nutrient balance.

In parallel, the application of AI-based modeling demonstrated that complex metabolomic datasets from *in vitro* fermentations can be classified with high accuracy. The models consistently identified ferulic acid, indole acetic acid, and related metabolites as key discriminators, providing candidate biomarkers of aleurone exposure. This dual approach not only strengthens mechanistic insight into aleurone’s functional role but also illustrates the value of AI as a complementary analytical tool for dietary evaluation.

Together, these findings highlight aleurone’s potential as a functional feed component in horses and establish a framework for integrating AI-driven metabolomic analysis into future *in vivo* validation and nutritional applications.

## Data Availability

The datasets presented in this study can be found in online reporitory. The name of the repository and accession number can be found below: https://figshare.com/, 10.6084/m9.figshare.30429076.
