# Supplementary material for: From wheat bran to equine gut: the in vitro fermentation dynamics of aleurone
Source: Front Physiol. 2025 Nov 11;16:1644738. doi: 10.3389/fphys.2025.1644738 (PMC12643889; doi:10.3389/fphys.2025.1644738)
Supplement: Supplementary file 1 [file Table1.docx]

Supplementary Material

# Supplementary Figures and Tables

## Supplementary Table

**Supplementary Table 1.**

Comparative feed analysis of the macronutrient composition of both the Blanco feed and the aleurone concentrate used in the study.

| **Blanco feed** | |
| --- | --- |
| **Raw materials** | **Amount (%)** |
| Wheat short flour | 29,982 |
| Barley | 21,000 |
| Soybeans 30% RC | 11,000 |
| Alfalfa for horses | 10,500 |
| Fine corn flakes RV 11 | 8,000 |
| Wheat nt. Grain (flour) | 6,525 |
| Molasses P67 | 4,000 |
| Palm oil | 2,475 |
| Fine chalk | 2,275 |
| Maize | 2,000 |
| Salt | 0,900 |
| Dry matter MD | 0,500 |
| Premix horse 0,25% | 0,400 |
| Sodium bicarbonate | 0,288 |
| MgO 90% | 0,125 |
| Vit E 50% | 0,030 |
| Total | 100,000 |
| **Nutrients** | **Amount in product** |
| Dry matter | 875,982 |
| Moisture | 124,018 |
| Crude ash | 77724,000 |
| Crude protein | 111,260 |
| Crude fat | 55,068 |
| Crude fiber | 105,091 |
| Other carbohydrates | 524,069 |
| Starch | 282,780 |
| Sugars | 59,396 |
| Sugars and starch | 342,123 |
| Horse Feed Unit | 825,987 |
| EWpa | 0,701 |
| FOS g/kg | 497,487 |
| Digestible crude protein | 85,562 |
| Lysine | 4,638 |
| Methionine | 1,789 |
| NSP g/kg | 312,479 |
| Na+ K-Cl meq/kg | 270,116 |
| Calcium | 12,449 |
| Potassium | 9,798 |
| Sodium | 4,505 |
| Chlorine | 6,408 |
| Magnesium | 2,627 |
| Total phosphorus | 4,315 |
| Selenium (mg/kg) | 0,544 |
| Sodium selenite (E8)/selenium | 0,544 |
| Iron (mg/kg) | 340,291 |
| Iron (II) sulfate monohydrate (E1)/iron | 200,000 |
| Manganese (mg/kg) | 119,176 |
| Manganese (II) oxide (E5)/manganese | 80,000 |
| Zinc (mg/kg) | 158,944 |
| Zinc oxide (E6)/zinc | 120,000 |
| Copper (mg/kg) | 39,504 |
| Copper (II) sulfate pentahydrate (E4)/copper | 34,000 |
| Iodine (mg/kg) | 1,059 |
| Anhydrous calcium iodate (E2)/iodine | 1,000 |
| Cobalt (mg/kg) | 0,891 |
| Cobalt (II) carbinate hydroxide (2:3) monohydrate | 0,000 |
| C18:2 g/kg | 14,567 |
| C18:3 g/kg | 1,327 |
| Vitamin A (E672) | 25000,000 |
| Vitamin D3 (E671) | 3000,000 |
| Vitamin E (All-rac-alpha-tocopheryl acetate) | 150,000 |
| Vitamin K3 | 4,000 |
| Vitamin B1 | 20,000 |
| Vitamin B2 | 25,000 |
| Calcium-D-Pantothenate | 30,000 |
| Vitamin B6 | 10,000 |
| Vitamin B9 | 4,000 |
| Vitamin B12 | 64,000 |
| Vitamin PP (Niacin) | 60,000 |
| Vitamin C | 0,000 |
| Biotin | 304,000 |

| **Aleurone concentrate** | |
| --- | --- |
| **Raw materials** | **Amount (%)** |
| Wheat aleurone | 20,000 |
| Barley | 20,045 |
| Soybeans 30% RC | 12,375 |
| Alfalfa for horses | 10,500 |
| Fine corn flakes RV 11 | 8,000 |
| Wheat short flour | 7,100 |
| Wheat nt. Grain (flour) | 4,775 |
| Molasses P67 | 4,000 |
| Palm oil | 2,425 |
| Fine chalk | 2,300 |
| Maize | 6,325 |
| Salt | 0,900 |
| Dry matter MD | 0,500 |
| Premix horse 0,25% | 0,400 |
| Sodium bicarbonate | 0,200 |
| MgO 90% | 0,125 |
| Vit E 50% | 0,030 |
| Total | 100,000 |
| **Nutrients** | **Amount in product** |
| Dry matter | 876,333 |
| Moisture | 123,667 |
| Crude ash | 76,651 |
| Crude protein | 113,603 |
| Crude fat | 54,997 |
| Crude fiber | 110,350 |
| Other carbohydrates | 525,339 |
| Starch | 260,134 |
| Sugars | 55,735 |
| Sugars and starch | 315,834 |
| Horse Feed Unit | 830,920 |
| EWpa | 0,708 |
| FOS g/kg | 493,734 |
| Digestible crude protein | 83,382 |
| Lysine | 4,567 |
| Methionine | 1,770 |
| NSP g/kg | 312,386 |
| Na+ K-Cl meq/kg | 255,069 |
| Calcium | 12,499 |
| Potassium | 10,423 |
| Sodium | 4,581 |
| Chlorine | 6,425 |
| Magnesium | 3,325 |
| Total phosphorus | 5,312 |
| Selenium (mg/kg) | 0,544 |
| Sodium selenite (E8)/selenium | 0,544 |
| Iron (mg/kg) | 340,559 |
| Iron (II) sulfate monohydrate (E1)/iron | 200,000 |
| Manganese (mg/kg) | 116,138 |
| Manganese (II) oxide (E5)/manganese | 80,000 |
| Zinc (mg/kg) | 157,778 |
| Zinc oxide (E6)/zinc | 120,000 |
| Copper (mg/kg) | 39,271 |
| Copper (II) sulfate pentahydrate (E4)/copper | 34,000 |
| Iodine (mg/kg) | 1,064 |
| Anhydrous calcium iodate (E2)/iodine | 1,000 |
| Cobalt (mg/kg) | 0,893 |
| Cobalt (II) carbinate hydroxide (2:3) monohydrate | 0,000 |
| C18:2 g/kg | 14,940 |
| C18:3 g/kg | 1,313 |
| Vitamin A (E672) | 25000,000 |
| Vitamin D3 (E671) | 3000,000 |
| Vitamin E (All-rac-alpha-tocopheryl acetate) | 150,000 |
| Vitamin K3 | 4,000 |
| Vitamin B1 | 20,000 |
| Vitamin B2 | 25,000 |
| Calcium-D-Pantothenate | 30,000 |
| Vitamin B6 | 10,000 |
| Vitamin B9 | 4,000 |
| Vitamin B12 | 64,000 |
| Vitamin PP (Niacin) | 60,000 |
| Vitamin C | 0,000 |
| Biotin | 304,000 |
