# Supplementary material for: From wheat bran to equine gut: the in vitro fermentation dynamics of aleurone
Source: Front Physiol. 2025 Nov 11;16:1644738. doi: 10.3389/fphys.2025.1644738 (PMC12643889; doi:10.3389/fphys.2025.1644738)
Supplement: Supplementary file 2 [file Presentation1.pdf]

FACULTEIT DIERGENEESKUNDE
accredited by EAEVE

ETHICAL COMMITTEE

Chairperson: Prof. dr. K. Hermans
Secretary: Prof. dr. C. Delesalle

E-mail secretarisec@UGent.be

Form for the ethical evaluation of animal research not considered as animal procedure by law

Reserved for the Ethical Committee on Animal Experiments:

Identification code of this application: From Wheat Bran to Equine Gut: The In Vitro Fermentation Dynamics of Aleurone
Date of approval: 11/06/2025

Name and signature chairperson and secretary of the ethical committee who confirm the approval:

Prof. Katleen Hermans

[redacted]

Prof. C. Delesalle

Title of the project/experiment:

From Wheat Bran to Equine Gut: The In Vitro Fermentation Dynamics of Aleurone

The undersigned scientist would like to request permission for using animals/parts of animals/animal derivatives for scientific purposes, not subjected to obligatory prior ethical approval by law (Belgian Royal Decree of 29 May 2013 and EU directive 2010/63/EU).
More precisely, I will be *(please tick the appropriate box)*:

UNIVERSITEIT GENT

☒ performing a scientific research with dead animals not euthanised for the purpose of the experiment or leftover animal materials (e.g. slaughterhouse materials, leftover blood samples, animal patients deceased or euthanised for reasons not induced by the current scientific research)

☐ performing a scientific research with dead animals euthanised for the purpose of the experiment, but who did not undergo any other procedure prior to the euthanasia by a legally accepted humane method (in such case: mention the method of euthanasia in the blue box below)

☐ performing a scientific research on live animals, but "below threshold", i.e. not likely to cause pain, suffering, distress or lasting harm equivalent to, or higher than, that caused by the introduction of a needle in accordance with good veterinary practice (e.g. behaviour monitoring in non-stressful conditions, treatment and follow-up of veterinary patients with no extra harm or discomfort than what the patient would undergo when treated normally according to good veterinary practice).

Please provide a short description (max. 150 words) of the experiment, showing that it belongs to the category selected above:

According to European Directive 2010/63/EU on the protection of animals used for scientific purposes, ethical approval is not required for studies involving materials obtained from animals that were not euthanized for the purpose of the experiment. In the present study, digesta from the jejunum, caecum, and colon of horses were collected ex vivo immediately after slaughter in a commercial slaughterhouse. These horses were not killed specifically for the purpose of this research, nor were they subjected to any experimental procedures prior to death. As such, the materials used qualify as "leftover animal material" and the study falls outside the scope of mandatory ethical approval. No living animals were used or harmed for the execution of the in vitro fermentation protocol, and all procedures were in accordance with current EU guidelines on the use of animal-derived material for scientific research.

I confirm to have completed this document truthfully.

Name scientist: Maarten Willems (PhD student) (Research Group of Comparative Physiology, Prof. C. elesalle)
Date: 11/06/2025
Signature:

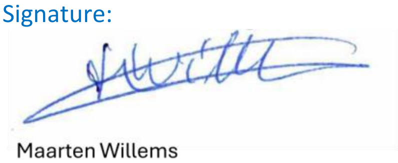

Background information from EU Directive 2010/63:

1) The definition of
   - ***a.*** a "procedure" on animals*: 'procedure' means any use, invasive or non-invasive, of an animal for experimental or other scientific purposes, with known or unknown outcome,*

or educational purposes, which may cause the animal a level of pain, suffering, distress or lasting harm equivalent to, or higher than, that caused by the introduction of a needle in accordance with good veterinary practice. This includes any course of action intended, or liable, to result in the birth or hatching of an animal or the creation and maintenance of a genetically modified animal line in any such condition, ***but excludes the killing of animals solely for the use of their organs or tissues.***

  b. a "project": *'project' means a program of work having a defined scientific objective and involving one or more procedures.*

2) The provisions for project authorisation:
   - *a.* *Member States shall ensure that projects are not carried out without prior authorization from the competent authority, and that projects are carried out in accordance with the authorization or in accordance with the application sent to the competent authority or any decision taken by the competent authority.*
   - *b.* *Member States shall ensure that no project is carried out unless a favorable project evaluation by the competent authority has been received in accordance with Article 38.*

3) The fact that the directive for the protection of animals used in experiments **does not apply to**:
   - ***a.*** ***non-experimental agricultural practices;***
   - ***b.*** ***non-experimental clinical veterinary practices;***
   - ***c.*** ***veterinary clinical trials required for the marketing authorization of a veterinary medicinal product;***
   - ***d.*** ***practices undertaken for the purposes of recognized animal husbandry;***
   - ***e.*** ***practices undertaken for the primary purpose of identification of an animal;***
   - ***f.*** ***practices not likely to cause pain, suffering, distress or lasting harm equivalent to, or higher than, that caused by the introduction of a needle in accordance with good veterinary practice.***
